# The Neglected Role of Physical Education Participation on Suicidal Ideation and Stress in High School Adolescents from South Korea

**DOI:** 10.3390/ijerph17082838

**Published:** 2020-04-20

**Authors:** Saengryeol Park, So-Youn Park, Su Yeon Jang, Gapjin Oh, In-Hwan Oh

**Affiliations:** 1 Department of Preventive Medicine, School of Medicine, Kyung Hee University, 26, Kyungheedae-ro, Dongdaemun-gu, Seoul 02453, Korea; saengryeol.park@gmail.com (S.P.); jcaecil@snu.ac.kr (S.Y.J.); 2 Department of Medical Education and Humanities, School of Medicine, Kyung Hee University, 26, Kyungheedae-ro, Dongdaemun-gu, Seoul 02453, Korea; ukii77@gmail.com; 3 Department of Sport Marketing, Kyung Dong University, 27, Kyundong University-ro, Yanju, Gyeonggido 11458, Korea; jim@kduniv.ac.kr

**Keywords:** Korea Youth Risk Behaviour Web-Based Survey, mental health, physical activity, youth, school health

## Abstract

Adolescents are at high risk of suicidal ideation and stress. This study aimed to investigate how physical education participation predicts suicidal ideation and stress in South Korean high school students. Data from the Twelfth Korea Youth Risk Behaviour Web-Based Survey 2016 (KYRBS) were used for analyses. Two multiple logistic regressions were performed to determine the influence of selected factors on suicidal ideation and stress (model 1: subjective health, social support, body mass index, academic achievement, perceived economic status of family, and physical education participation; model 2: adjusting for school type and year). Model 2 revealed negative associations between subjective health, academic achievement, perceived economic status of family, social support, physical education participation (≥2 times/weekly), and suicidal ideation for male students. Female students exhibited negative associations between subjective health, social support, and academic achievement, along with a positive association between body mass index and suicidal ideation. For both genders, stress was negatively associated with subjective health, social support, academic achievement, perceived economic status of family, and physical education participation (≥2 times/weekly). These findings suggest that participating in physical education can mitigate the risk of suicidal ideation and stress among high school students.

## 1. Introduction

Mental health problems constitute a high proportion (16%) of global disease and injury burden among adolescents [1]. In South Korea, such problems are projected to become the third leading disease group of disability-adjusted life years among adolescents by 2030 [2]. Psychological problems in this country tend to begin during adolescence (≥14 years) [1] and are diagnosed at a young age (≤25 years) [3]. In 2016, 42.4% of adolescents experienced high stress, with suicide being the greatest cause of mortality [4]. Given that suicidal ideation could be an early contributing factor of suicide attempts [5], an urgent investigation is needed to cope with the spread of suicidal ideation and stress.

Factors linked to suicidal ideation and stress in adolescents are well established at the intrapersonal, interpersonal, and community levels. For example, being male, having good physical health [6], social support [7], low body mass index [8], and living in rural areas [9] are associated with decreased suicidal ideation. Social support from family and high academic achievement are associated with less stress [10], while subjective health [11] and high body weight [12] are linked to high stress. Importantly, increased physical activity is correlated with low suicidal ideation [13] and stress [14,15]. Despite the possible benefits, many high school students are less likely to participate in physical activity outside of school (vigorous physical activity = 27.26 min/week, moderate physical activity = 36.99 min/week) [16]. However, when adolescents participated in high levels of physical education at school, they also exhibited elevated physical activity outside of school [17]. This link implies the need to verify the potential role of physical education on adolescent mental health. 

A number of beneficial associations between physical education and mental health have been reported [18]. For example, students who engaged in frequent physical education also had high academic achievement [19]. In addition, increased participation in physical education is associated with a lower likelihood of obesity and cardiovascular risk among adolescents [20]. Likewise, when adolescents have good experiences with their physical education, they may accumulate a positive attitude towards physical activity generally [21]. Positive interactions with teachers could also affect health-related quality of life [22]. Therefore, these classes are potentially an important setting to promote values (e.g., active lifestyles) that discourage suicidal ideation and stress. However, as far as we are aware, little is known regarding the role of physical education in influencing suicidal ideation and stress among adolescents.

Available research on physical education has largely focused on improvements to leisure-time physical activity and physical health [23,24,25]. Although a study found no associations between physical education and suicidal behaviors in middle school students [26], high educational pressure forces adolescents to spend about 15 h a day on studying, including school, homework, and private tutoring (cram schools) [27]. This heavy workload may lead to elevated stress levels. Despite the compulsory nature of physical education in South Korea [28], approximately 75% of high schools failed to implement national curriculum guidelines for physical education (150 min/week) [29], instead replacing that time with self-guided study to prepare for university admission exams [28]. Promoting awareness of physical activity’s potential ameliorating effects is one strategy to proactively cope with mental health problems in adolescents. The aim of this research was to verify whether participating in physical education influences suicidal ideation and stress in high school students from South Korea. Because physical activity participation has a clear gender difference [16], we examined male and female students separately.

## 2. Materials and Methods

### 2.1. Participants and Procedures

**Figure 1:**
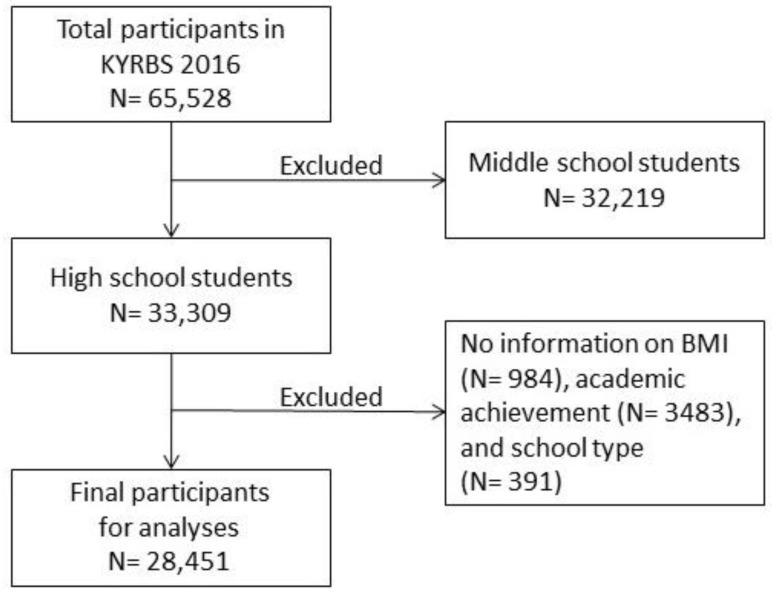
Flow chart of participant selection. KYRBS = the Twelfth Korea Youth Risk Behaviour Web-Based Survey 2016, BMI = body mass index.

In total, 65,528 participants were included in the Twelfth Korea Youth Risk Behaviour Web-Based Survey 2016 (KYRBS; Figure 1). Only high school students were included in the current analysis. Participants with missing data on body mass index (BMI), academic achievement, and school type were excluded to yield a final sample size of 28,451. 

The current cross-sectional study was conducted from October 2019 to February 2020 using the secondary data obtained from KYRBS [30] after ethics approval by the University’s Institutional Review Board (IRB No. KHSIRB-19-354 (EA)). KYRBS, which is an internet-based and self-administered national survey, was set up to monitor school health and has been managed by South Korea’s Centers for Disease and Prevention (CDC) since 2005. KYRBS uses a multi-stage cluster sampling design to collect data on health-risk behaviors in South Korean adolescents, including smoking status, alcohol, obesity, diet, and physical activity [31]. During the data collection of KYRBS, students did not provide any personal identifiers such as names, phone numbers, or living address. Participating students were all volunteers across 400 middle and high schools, representing 17 administrative areas. Students took the survey in their school’s computer room under teacher supervision and their anonymity was ensured. They provided consent before the survey was initiated [32]. Detailed procedures are published elsewhere [31].

### 2.2. Variables

#### 2.2.1. Subjective Health

Subjective health is an individual’s own perception of overall or physical health status [33]. Subjective health was assessed with a one-item scale (“How would you rate your health in general?”) on a five-point Likert scale (“very unhealthy” = 1 to “very healthy” = 5) [34]. High scores indicate high subjective health.

#### 2.2.2. Social Support

Social support is the emotional or tangible support from interpersonal relationships; evidence suggests it modulates physical and mental health [35]. Social support was assessed with a one-item scale (“Can you talk about your concerns with people (i.e., father, mother, brothers, sisters, friends, teachers, and others)?”). Answers were dichotomized (no = 0 and yes = 1) [36].

#### 2.2.3. Body Mass Index

To calculate BMI (kg/m^2^), weight (kg) is divided by the square of height (m). This index is a rough measure of body fat and is widely used to categorize individuals as normal weight, overweight, or obese [37]. BMI was estimated via self-reported height and weight.

#### 2.2.4. Academic Achievement

Academic achievement, or how well a student performs in school, affects socio-economic status, accessibility of medical care, and occupational choices [38]. As students are aware of these long-term outcomes, scholastic performance may influence their mental health. Academic achievement was assessed with one item (“How was your grade point average in the last 12 months?”) and recorded on a five-point Likert scale (low = 1, mid-low = 2, mid = 3, mid-high = 4, high = 5) [39]. The scale was converted into a three-level variable (low/mid-low = 1, mid = 2, mid-high, high = 3) [40]. High scores indicate high academic achievement.

#### 2.2.5. Participation in Physical Education

Physical education is the planned instruction of exercises and other physical activity during school hours [41]. Physical education participation was measured using one item (“In an average week when you are in school, on how many days do you exercise in physical education classes?”) on a four-point Likert scale (0, 1, 2, ≥3 times/week) [23]. The scale was converted into a three-level variable (0, 1, ≥2 times/week).

#### 2.2.6. Residential Area

Participant living environment was categorized into rural = 0 and urban = 1. Rural areas included countries and urban areas included metropolitan cities.

#### 2.2.7. Suicidal Ideation

Suicidal ideation is “thinking about, considering, or planning suicide” [42] and was assessed using one item (“During the past 12 months, did you ever seriously think about committing suicide?”) [27]. The answer was recorded as “no” = 0 or “yes” = 1.

#### 2.2.8. Stress

Stress is the anxiety, discomfort, and emotional tension that occurs after perceiving a threat or other difficult situation [43]. Stress was measured with one item (“How often do you feel stress in your typical daily life?”) on a five-point Likert scale (“not at all” = 1, “not so much” = 2, “a little bit” = 3, “frequently” = 4,” very frequently” = 5) [39]. The answer was categorized as “not stressed = 0 (1, 2, 3)” or “stressed = 1 (4, 5)” [43].

#### 2.2.9. Demographics

A range of demographic information was collected, including age and perceived household economic status. The latter was assessed using one item (“How do you perceive your household economic status?”) on a five-point Likert scale (low = 1 to high = 5) [43]. The answer was transformed into three categories (low = 1, medium = 2, high = 3). Higher scores indicate better economic status. In KYRBS, high schools were categorized into academic and vocational [44]; this was coded in the current analysis as (“general high school” = 0 vs. “vocational high school” = 1. The former’s primary purpose is to improve academic performance; these include general, autonomous, science, foreign language, art, sport, and international high schools. The latter aims to provide field experience and includes agriculture/life industry, engineering, commercial information, fishery, and business high schools. School year (years 1, 2, and 3) were also measured.

### 2.3. Statistical Analysis

Descriptive statistics were performed to examine participant characteristics. Multiple logistic regressions were performed to investigate the associations of predictors with suicidal ideation, happiness, and stress. Regression models were conducted in two steps: (1) with no adjustment and (2) adjusting for school type and school year. Analyses were conducted in SPSS version 23 (SPSS Inc., Armonk, NY, USA) and statistical significance was set at *p* = 0.05.

## 3. Results

### 3.1. Participant Characteristics

The final analysis included 28,451 students (male = 14,263, mean age = 16.41; female = 14,188, mean age = 16.42) (Table 1). On average, male students had slightly higher subjective health and BMI than female students. Female students reported a higher proportion of social support than male students. Overall, more students had social support than not. Most students resided in urban settings and attended general high schools instead of vocational high schools. Most also perceived their household economic status to be in the middle.

### 3.2. Predictors of Suicidal Ideation

Logistic regression model 1 (Table 2) indicated that subjective health, academic achievement, and perceived household economic status were significantly associated with a decreased likelihood of suicidal ideation in both genders. Social support was significantly associated with a lower likelihood of suicidal ideation for all students. Among girls, BMI was significantly associated with a greater likelihood of suicidal ideation. These associations persisted even after adjusting for school type and school year (model 2). Physical education at least twice a week was significantly associated with a lower likelihood of suicidal ideation, but only among boys. When examining covariates, school type was associated with suicidal ideation, also only in boys.

### 3.3. Predictors of Stress

In logistic regression model 1 (Table 3), subjective health, academic achievement, perceived household economic status, and BMI were significantly associated with a decreased likelihood of stress in both genders. Similarly, social support and physical education for more than twice a week were significantly associated with a lower likelihood of stress for all students. These associations (except BMI) persisted even after adjusting for school type and school year. School type was associated with a greater likelihood of stress for male students, while school year and increased stress were linked for both genders.

## 4. Discussion

We aimed to investigate intrapersonal, interpersonal, school, and community-level factors that predict suicidal ideation and stress using nationally representative data from South Korean adolescents. Physical education was associated with a decreased likelihood of suicidal ideation and stress, although this effect was dependent on gender and participation frequency. 

Previous studies have identified effects of intrapersonal, interpersonal, and environmental factors on suicidal ideation [6,7,8,9]. Here, we add to that existing body of knowledge by demonstrating that physical education (≥2 times/weekly) may reduce suicidal ideation in male high school students. Our findings partially corroborate earlier research showing that physical activity outside of school is linked to lower suicidal ideation [13]. However, our data contradicted a previous study showing that physical education was not associated with suicidal ideation among middle school students [26]. Interestingly, that same study showed that sports team engagement inside or outside school is linked to a decreased likelihood of suicidal ideation [26]. The apparent benefit of sports teams may be due to increased freedom (choice to attend), given that these sports are outside regular school curricula. Another possible reason for the difference between that study and ours is the participant pool; middle school students may experience less academic pressure than high school students. Finally, the previous study did not investigate gender differences, and so the lack of an effect among girls may have obscured an effect among boys. Following the national curriculum on physical education and providing suitable prevention programs therein could alleviate suicidal ideation and potentially reduce suicide attempts among male students [5].

The lack of association between physical education and suicidal ideation in female students may be partly attributable to lower interest among girls in physical education due to self-consciousness, low motivation [45], and competence [46,47]. However, suicidal ideation rates are higher in female than in male adolescents [48], so further research is urgently warranted to determine how educators can encourage more participation in physical education among young women.

Our results suggest that physical education is potentially an effective method to alleviate stress among high school students, in line with previous qualitative research conducted in the United States [49]. Stress levels among South Korean high school students are very high because of increased academic pressure [50] and greater emphasis on education [39] compared with other cultures. Indeed, two thirds of adolescents suffer from stress in South Korea [4]. Therefore, when attempting to implement curriculum guidelines for physical education, schools should exercise caution to avoid performance stress in the event of that students do poorly in physical education classes [51]. Importantly, physical education should not be added on top of existing academic classes; instead, high schools could instead reallocate self-guided study time to physical education [28].

Developing the interpersonal skills of physical education teachers may be necessary to incorporate a variety of programs that could encourage participation from male students [52]. For example, programs could provide a wider range of physical activities that interest students and increase their sense of accomplishment. In addition to lowering stress, physical education can also increase peer inclusion [51].

Other barriers to physical education that should be addressed include insufficient quantity and quality of facilities [28] and the recent coronavirus disease-19 pandemic. Adolescents may be reluctant to participate because of outbreak-induced anxiety and perceived difficulties in social distancing during physical activity [53]. Thus, educational professionals are encouraged to expand infrastructure related to physical activity (e.g., gymnasiums), as well as to develop teaching programs that stimulate participation while attending to student mental health.

The cross-sectional nature of our study means we could not infer any causation for suicidal ideation and stress, a limitation that future longitudinal or experimental designs should address. Second, although physical education predicted suicidal ideation (in boys only) and stress (both genders), there is no data on whether the association extends to suicidal attempts [5]. Further studies could address this question via mediation or structural analyses. Finally, the results of the current study are not fully generalizable because we used self-reports to measure participation in physical education. To lessen measurement bias, accelerometers are recommended as an objective method to assess the duration and intensity of physical activity [54]. We do note the value of using a database such as KYRBS for a nationally representative assessment; moreover, the results of the current study can be easily compared with previous adolescent research that also employed KYRBS data [16].

## 5. Conclusions

This study used nationally representative data on South Korean high school students to demonstrate that physical education predicts lower suicidal ideation and stress, even after accounting for well-known covariates. Currently, only a quarter of high schools successfully implement national curriculum guidelines for physical education [29], which is approximately 170 h/year [55]. We therefore conclude that there is a clear need to increase participation in physical education. In addition, we recommend more research on interventions to promote such participation in South Korean high school students to improve their overall psychological health.

## Figures and Tables

**Table 1:** Characteristics of students (N = 28,451).

Characteristic	Male (N = 14,263)	Female (N = 14,188)
Age (years)	16.41 (0.95)	16.42 (0.95)
Subjective health	3.96 (0.86)	3.66 (0.86)
Existence of social support, N (%)		
Yes	10,539 (73.89)	12,562 (88.54)
No	3724 (26.11)	1626 (11.46)
Body mass index, mean (SD)	22.15 (3.60)	21.24 (2.83)
Academic achievement, N (%)		
Low	1927 (13.51)	1440 (10.15)
Mid	7692 (53.93)	7840 (55.26)
High	4644 (32.56)	4908 (34.59)
Residential area, N (%)		
Rural	1061 (7.44)	975 (6.87)
Urban	13,202 (92.56)	13,213 (93.13)
School type, N (%)		
General high school	11,278 (79.07)	11,829 (83.37)
Vocational high school	2985 (20.93)	2359 (16.63)
Perceived household economic status, N (%)		
Low	568 (3.98)	478 (3.37)
Mid	12,833 (89.97)	13,165 (92.79)
High	862 (6.04)	545 (3.84)
Suicidal ideation, N (%)		
Yes	1315 (9.22)	2068 (14.58)
No	12,948 (90.78)	12,120 (85.42)
Stress, N (%)		
Yes	4572 (32.05)	7009 (49.40)
No	9691 (67.95)	7179 (50.60)

**Table 2:** Predictors of suicidal ideation (N = 28,451).

Predictors	Male						Female					
	Model 1			Model 2			Model 1			Model 2		
	ORs	95% CI	*p*-Value	ORs	95% CI	*p*-Value	ORs	95% CI	*p*-Value	ORs	95% CI	*p*-Value
Constant	1.836	-	0.045	2.029	-	0.046	2.151	-	0.011	2.749	-	0.003
Subjective health	0.630	0.591–0.672	<0.001	0.630	0.591–0.672	<0.001	0.610	0.577–0.645	<0.001	0.608	0.575–0.643	<0.001
Social support	0.663	0.587–0.749	<0.001	0.665	0.589–0.752	<0.001	0.440	0.389–0.498	<0.001	0.441	0.389–0.499	<0.001
BMI	1.002	0.987–1.017	0.815	1.003	0.987–1.018	0.728	1.030	1.013–1.047	<0.001	1.031	1.014–1.048	<0.001
Academic achievement	0.813	0.745–0.887	<0.001	0.815	0.747–0.889	<0.001	0.780	0.723–0.842	<0.001	0.782	0.725–0.844	<0.001
Perceived economic status	0.802	0.669–0.962	0.018	0.783	0.652–0.941	0.009	0.850	0.713–1.014	0.071	0.842	0.706–1.005	0.057
Residential area	1.128	0.898–1.418	0.301	1.075	0.854–1.355	0.538	1.078	0.887–1.310	0.450	1.078	0.887–1.310	0.452
Physical education participation												
0 day	(reference)											
1 day	0.874	0.727–1.052	0.154	0.882	0.733–1.061	0.184	1.077	0.942–1.232	0.278	1.087	0.950–1.244	0.227
≥2 days	0.865	0.737–1.016	0.077	0.850	0.723-0.999	0.048	1.096	0.967–1.242	0.153	1.079	0.951–1.224	0.241
Covariates												
School type	-	-	-	1.234	1.062–1.434	0.006	-	-	-	1.045	0.918–1.190	0.504
School year	-	-	-	0.961	0.894–1.034	0.288	-	-	-	0.948	0.892–1.007	0.081

BMI = body mass index, ORs = odds ratio, CI = confidence interval.

**Table 3:** Predictors of stress (N = 28,451).

Predictors	Male						Female					
	Model 1			Model 2			Model 1			Model 2		
	ORs	95% CI	*p*-Value	ORs	95% CI	*p*-Value	ORs	95% CI	*p*-Value	ORs	95% CI	*p*-Value
Constant	7.525	-	<0.001	4.456	-	<0.001	18.801	-	<0.001	11.852	-	<0.001
Subjective health	0.630	0.604–0.657	<0.001	0.629	0.603–0.657	<0.001	0.568	0.545–0.593	<0.001	0.570	0.546–0.594	<0.001
Social support	0.716	0.661–0.776	<0.001	0.715	0.660–0.775	<0.001	0.514	0.459–0.576	<0.001	0.513	0.458–0.574	<0.001
BMI	1.010	1.000–1.020	0.042	1.009	0.999–1.019	0.082	1.022	1.010–1.035	<0.001	1.021	1.009–1.034	<0.001
Academic achievement	0.832	0.787–0.879	<0.001	0.832	0.787–0.879	<0.001	0.846	0.800–0.894	<0.001	0.842	0.797–0.890	<0.001
Perceived economic status	0.829	0.737–0.932	0.002	0.815	0.724–0.917	<0.001	0.862	0.756–0.982	0.026	0.868	0.761–0.989	0.034
Residential area	0.933	0.814–1.070	0.321	0.889	0.774–1.021	0.096	0.979	0.855–1.121	0.757	0.968	0.845–1.109	0.639
Physical education participation												
0 day	(reference)											
1 day	0.913	0.809–1.031	0.142	0.906	0.802–1.024	0.113	0.963	0.874–1.060	0.438	0.947	0.859–1.043	0.268
≥2 days	0.871	0.784–0.969	0.011	0.879	0.789–0.978	0.018	0.878	0.802–0.960	0.004	0.895	0.818–0.980	0.016
Covariates												
School type	-	-	-	1.278	1.165–1.402	<0.001	-	-	-	1.044	0.951–1.145	0.365
School year	-	-	-	1.092	1.043–1.143	<0.001	-	-	-	1.092	1.046–1.141	<0.001

BMI = body mass index, ORs = odds ratio, CI = confidence interval.

## References

[1] World Health Organization (2019). Adolescent Mental Health. https://www.who.int/news-room/fact-sheets/detail/adolescent-mental-health.

[2] Park B., Park B., Han H., Choi E.J., Kim N., Shin Y., Park H. (2019). Projection of the years of life lost, years lived with disability, and disability-Adjusted life years in Korea for 2030. J. Korean Med. Sci..

[3] Kessler R.C., Berglund P., Demler O., Jin R., Merikangas K.R., Walters E.E. (2005). Lifetime prevalence and age-Of-Onset distributions of dsm-Iv disorders in the national comorbidity survey replication. Arch. Gen. Psychiatry.

[4] Statistics Korea (2019). Statistics Korea Adolescent Statistics. http://kostat.go.kr/portal/korea/kor_nw/1/6/1/index.board?bmode=read&aSeq=374490&pageNo=&rowNum=10&amSeq=&sTarget=&sTxt=.

[5] Klonsky E.D., May A.M., Saffer B.Y. (2016). Suicide, suicide attempts, and suicidal ideation. Ann. Rev. Clin. Psychol..

[6] Bridge J.A., Goldstein T.R., Brent D.A. (2006). Adolescent suicide and suicidal behavior. J. Child. Psychol. Psychiatry.

[7] King C.A., Merchant C.R. (2008). Social and interpersonal factors relating to adolescent suicidality: A review of the literature. Arch. Suicide Res..

[8] Eaton D.K., Lowry R., Brener N.D., Galuska D.A., Crosby A.E. (2005). Associations of body mass index and perceived weight with suicide ideation and suicide attempts among US high school students. Arch. Pediatr. Adol. Med..

[9] Goldman-Mellor S., Allen K., Kaplan M.S. (2018). Rural/urban disparities in adolescent nonfatal suicidal ideation and suicide attempt: A population-Based study. Suicide Life Threat. Behav..

[10] Park S., Jang H., Lee E.-S. (2018). Major stressors among Korean Adolescents according to gender, educational level, residential area, and socioeconomic status. Int. J. Environ. Res. Public Health.

[11] Moksnes U.K., Espnes G.A. (2017). Stress, sense of coherence and subjective health in adolescents aged 13–18 years. Scand. J. Public Health.

[12] Hamaideh S.H., Al-Khateeb R.Y., Al-Rawashdeh A.B. (2010). Overweight and obesity and their correlates among jordanian adolescents. J. Nurs. Scholarsh..

[13] Vancampfort D., Hallgren M., Firth J., Rosenbaum S., Schuch F.B., Mugisha J., Probst M., Van Damme T., Carvalho A.F., Stubbs B. (2018). Physical activity and suicidal ideation: A systematic review and meta-Analysis. J. Affect. Disord..

[14] Kim H.J., Oh S.Y., Lee D.W., Kwon J., Park E.-C. (2019). The effects of intense physical activity on stress in adolescents: Findings from Korea Youth Risk Behavior Web-Based Survey (2015–2017). Int. J. Environ. Res. Public Health.

[15] Moljord I.E., Moksnes U.K., Eriksen L., Espnes G.A. (2011). Stress and happiness among adolescents with varying frequency of physical activity. Percep. Mot. Ski..

[16] Sok S.A., Kim S.M., Jung J.H., An S.Y., Kang H.E. (2017). Relationship between physical activity and subjective quality of sleep in Korean high school students, The 11th Korea Youth Risk Behavior Web-Based Survey. Korean J. Fam. Pract..

[17] Pate R.R., O’Neill J.R., McIver K.L. (2011). Physical activity and health: Does physical education matter?. Quest.

[18] Zach S., Shoval E., Lidor R. (2017). Physical education and academic achievement—Literature review 1997–2015. J. Curric. Stud..

[19] Rasberry C.N., Lee S.M., Robin L., Laris B.A., Russell L.A., Coyle K.K., Nihiser A.J. (2011). The association between school-Based physical activity, including physical education, and academic performance: A systematic review of the literature. Prev. Med..

[20] Hohensee C.W., Nies M.A. (2017). Physical activity in American schools and body mass index percentile. J. Child. Health Care.

[21] Washington R.L. (2009). Physical education in schools helps reduce future cardiovascular risk. Circulation.

[22] Silverman S. (2005). Thinking long term: Physical education’s role in movement and mobility. Quest.

[23] Morgan C.F., Beighle A., Pangrazi R.P. (2007). What are the contributory and compensatory relationships between physical education and physical activity in children?. Res. Quart Exer. Sport.

[24] Bailey R., Armour K., Kirk D., Jess M., Pickup I., Sandford R. (2009). BERA Physical Education and Sport Pedagogy Special Interest Group. The educational benefits claimed for physical education and school sport: An academic review. Res. Papers Educ..

[25] Kleppang A.L., Hartz I., Thurston M., Hagquist C. (2019). Leisure-Time physical activity among adolescents and subsequent use of antidepressant and hypnotic drugs: A prospective register linkage study. Eur. Child. Adolesc. Psychiatry.

[26] Southerland J.L., Zheng S., Dula M., Cao Y., Slawson D.L. (2016). Relationship between physical activity and suicidal behaviors among 65,182 middle school students. J. Phys. Act. Health.

[27] ICEF Monitor (2014). High Performance, High Pressure in South Korea’s Education System—ICEF Monitor—Market Intelligence for International Student Recruitment. https://monitor.icef.com/2014/01/high-performance-high-pressure-in-south-koreas-education-system/.

[28] Lee K.C., Cho S.M. (2014). The Korean national curriculum for physical education: A shift from edge to central subject. Phys. Educ. Sport Pedagog..

[29] Yoon Y.H. (2019). To Prevent the Vicious Circle of Student Health School Should Be a Place of Health Management. Chosunedu.

[30] Ministry of Education, Ministry of Health and Welfare, & Korea Centers for Disease Control and Prevention (2016). The Twelfth Korea Youth Risk Behavior Web-Based Survey. https://yhs.cdc.go.kr/new/pages/pds1.asp.

[31] Kim Y., Choi S., Chun C., Park S., Khang Y.-H., Oh K. (2016). Data resource profile: The Korea Youth Risk Behavior Web-Based Survey (KYRBS). Int. J. Epidemiol..

[32] Korea Centers for Disease Control and Prevention Korea Youth Risk Behviour Web-Based Survey. http://www.cdc.go.kr/yhs/.

[33] Monden C., Michalos A.C. (2014). Subjective health and subjective well-Being. Encyclopedia of Quality of Life and Well-Being Research.

[34] Jaelim C., Cho S.-K., Cho J., Cho J. (2016). Association between perceived stress and asthma symptoms in adolescents. Arch. Comm. Med. Public Health.

[35] Lakey B., Cohen S., Cohen S., Underwood L.G., Gottlieb B.H. (2000). Social support and theory. Social Support Measurement and Intervention: A Guide for Health and Social Scientists.

[36] Bang M.H., Yang S. (2018). Factors influencing depression and suicide attempts among South Korean juvenile victims of violence: Secondary data analysis from the 11th Korea Youth Risk Behavior Web-Based Survey. J. Korean Acad. Psychiatr. Mental Health Nurs..

[37] World Health Organization Obesity. https://www.who.int/topics/obesity/en/.

[38] Burrows T., Goldman S., Pursey K., Lim R. (2017). Is there an association between dietary intake and academic achievement: A systematic review. J. Hum. Nutr. Diet..

[39] So E.S., Park B.M. (2016). Health behaviors and academic performance among Korean adolescents. Asian Nurs. Res..

[40] Lee J., Kim T.H., Min S., Kim M.H., Park K.C., Moon J.S., Ahn J.S. (2018). Depressive symptoms and suicidal behaviors in adolescent non-Daily smokers compared to daily smokers and never-smokers in Korea: National cross-Sectional study. PLoS ONE.

[41] Perth & Kinross Council. https://www.pkc.gov.uk/media/16051/Definitions-of-Physical-Education/pdf/Definitions_of_Physical_Education.pdf?m=636110986627130000.

[42] Selye H. (1976). Stress in Health and Disease.

[43] Min J.H., Lee E.-Y., Spence J.C., Jeon J.Y. (2017). Physical activity, weight status and psychological well-Being among a large national sample of South Korean adolescents. Mental Health Phys. Activ..

[44] Ministry of Education Secondary Education. http://english.moe.go.kr/sub/info.do?m=020103&s=english.

[45] Biddle S.J., Coalter F., O’Donova T., MacBeth J., Nevill M., Whitehead S. Increasing Demand for Sport and Physical Activity by Girls. http://www.paha.org.uk/Resource/increasing-demand-for-sport-and-physical-activity-by-girls.

[46] Inchley J., Kirby J., Currie C. (2011). Longitudinal changes in physical self-perceptions and associations with physical activity during adolescence. Pediatr. Exer. Sci..

[47] Institute of Youth Sport (1999). The Girls in Sport Project: Interim Report.

[48] Im Y., Oh W.-O., Suk M. (2017). Risk factors for suicide ideation among adolescents: Five-Year national data analysis. Arch. Psychiatr. Nurs..

[49] Howard H.Z., Hipscher M., Leung R.W., Zeng H.Z., Hipscher M., Leung R.W. (2011). Attitudes of high school students toward physical education and their sport activity preferences. J. Soc. Sci..

[50] Lee M., Larson R. (2000). The Korean ‘Examination Hell’: Long hours of studying, distress, and depression. J. Youth Adol..

[51] Lyngstad I., Bjerke Ø., Lagestad P. (2020). Students’ views on the purpose of physical education in upper secondary school. Physical education as a break in everyday school life–learning or just fun?. Sport Educ. Soc..

[52] Ntoumanis N., Quested E., Reeve J., Cheon S.H., Jackson B., Dimmock J., Compton J. (2017). Need-Supportive communication: Implications for motivation in sport, exercise, and physical activity. Persuasion and Communication in Sport, Exercise, and Physical Activity.

[53] World Health Organization Obesity. https://www.who.int/emergencies/diseases/novel-coronavirus-2019/.

[54] Colley R.C., Butler G., Garriguet D., Prince S.A., Roberts K.C. (2019). Comparison of self-Reported and accelerometer-Measured physical activity among Canadian youth. Health Rep..

[55] The Ministry of Education General guidelines of the curriculum, The Ministry of Education Guidelines. http://www.law.go.kr/LSW/admRulInfoP.do?admRulSeq=2100000141712.

